# Pretreatment biopsy analysis of DAB2IP identifies subpopulation of high‐risk prostate cancer patients with worse survival following radiation therapy

**DOI:** 10.1002/cam4.554

**Published:** 2015-10-16

**Authors:** Corbin Jacobs, Vasu Tumati, Payal Kapur, Jingsheng Yan, Xian‐Jin Xie, Raquibul Hannan, Jer‐Tsong Hsieh, Dong Wook Nathan Kim, Debabrata Saha

**Affiliations:** ^1^ Department of Radiation OncologyUniversity of Texas Southwestern Medical CenterDallasTexas75390; ^2^ Department of PathologyUniversity of Texas Southwestern Medical CenterDallasTexas75390; ^3^ Simmons Cancer CenterUniversity of Texas Southwestern Medical CenterDallasTexas75390; ^4^ Department of UrologyUniversity of Texas Southwestern Medical CenterDallasTexas75390

**Keywords:** Biomarker, DAB2IP, EZH2, prostate cancer, survival

## Abstract

Decreased expression of tumor suppressor DAB2IP is linked to aggressive cancer and radiation resistance in several malignancies, but clinical survival data is largely unknown. We hypothesized that pretreatment DAB2IP reduction would predict worse prostate cancer‐specific survival (PCSS). Immunohistochemistry of pretreatment biopsies was scored by an expert genitourinary pathologist. Other endpoints analyzed include freedom from biochemical failure (FFBF), castration resistance‐free survival (CRFS), and distant metastasis‐free survival (DMFS). Seventy‐nine patients with NCCN‐defined high‐risk prostate cancer treated with radiotherapy from 2005 to 2012 at our institution were evaluated. Twenty‐eight percent (22/79) of pretreatment biopsies revealed DAB2IP‐reduction. The median follow up times were 4.8 years and 5.3 years for patients in the DAB2IP‐reduced group and DAB2IP‐retained group, respectively. Patients with reduced DAB2IP demonstrated worse outcome compared to patients retaining DAB2IP, including FFBF (4‐year: 34 vs. 92%; *P* < 0.0001), CRFS (4‐year: 58 vs. 96%; *P* = 0.0039), DMFS (4‐year: 58 vs. 100%; *P* = 0.0006), and PCSS (5‐year: 83 vs. 100%; *P* = 0.0102). Univariate analysis showed T stage, N stage, and Gleason score were statistically significant variables. Pretreatment tumor DAB2IP status remained significant in multivariable analyses. This study suggests that about one‐fourth of men with high‐risk prostate cancer have decreased tumor expression of DAB2IP. This subpopulation with reduced DAB2IP has a suboptimal response and worse malignancy‐specific survival following radiation therapy and androgen deprivation. DAB2IP loss may be a genetic explanation for the observed differences in aggressive tumor characteristics and radiation resistance. Further study into improving treatment response and survival in this subpopulation is warranted.

## Introduction

Prostate cancer is the most common male malignancy and the second leading cause of cancer‐related death in the United States with 220,800 cases and 27,540 deaths expected in 2015 1, of which 20–30% have high‐risk features 2. For high‐risk prostate cancer defined by the National Comprehensive Cancer Network (NCCN) as Stage ≥cT3a, Gleason score ≥8, or prostate‐specific antigen (PSA) ≥20, standard treatment guidelines include definitive radiation therapy with neoadjuvant, concurrent, and long‐term androgen deprivation therapy in the United States 3. In spite of standard therapy, long‐term outcomes are suboptimal with 5‐year biochemical progression‐free survival of 60–70% and 5‐year overall survival of 75–85% 4, 5, 6, 7. Synthesizing all this data, one in four to five men with prostate cancer is diagnosed with high‐risk disease and despite treatment for their locally advanced malignancy, roughly 30–40% of these men suffer a relapse which accounts for most prostate cancer deaths.

Being able to predict the patients that portend worse outcome at the time of diagnosis may provide means to offer patients an alternative, more effective therapy. Biomarkers that can readily identify patients most likely to fail conventional therapy is warranted, and investigations of the molecular pathway involved in this poor outcome may further lead to future therapeutic interventions. One such potential biomarker is DOC‐2/DAB2 interactive protein (DAB2IP).

DAB2IP, a member of the RAS‐GAP protein family, is a novel and putative tumor suppressor. DAB2IP protein is a potent growth inhibitor by inducing G(0)/G(1) cell cycle arrest and is proapoptotic in response to stress 8. Decreased expression of DAB2IP has been associated with aggressive prostate cancer and radiation resistance in cell culture models 8, 9, 10, rats 11, and human tumors 12, 13. The mechanism underlying radiation resistance of DAB2IP‐deficient tumors can be explained by elevated ATM expression and activation, increased S phase cell distribution, accelerated DNA double‐strand break repair kinetics, evasion of apoptosis, and increased autophagy following ionizing radiation 8, 9, 10, 14. Loss of DAB2IP expression initiates epithelial‐to‐mesenchymal transition which leads to multiple lymph node and distant organ metastases 15. Other than radiation resistance, loss of DAB2IP may promote castration resistance 13. This is because one of DAB2IP's functions is to inhibit androgen receptor‐mediated cell growth and gene activation in prostate cells via both androgen‐dependent and androgen‐independent mechanisms 16.

Decreased expression of DAB2IP is often detected in aggressive prostate cancer cells, and this loss of DAB2IP is primarily due to altered epigenetic regulation of its promoter particularly by histone acetylation 17, 18. *DAB2IP* promoter methylation is frequently present in human breast cancer as well which plays a key role in DAB2IP inactivation and lymph node metastasis 19. Other than breast and prostate cancer, decreased DAB2IP expression by promoter methylation has been identified in several other malignancies including hepatocellular carcinoma 20, lung cancer 21, and gastrointestinal tumors 22. Another recently identified mechanism leading to the loss of DAB2IP protein is proteasome degradation mediated by oncogenic S‐phase kinase‐associated protein‐2 (Skp2) 23.

DAB2IP is also down‐regulated in bladder cancer with aggressive phenotypes 24. DAB2IP‐knockdown of bladder cancer cells by siRNA exhibit increased clonogenic survival in response to ionizing radiation compared with control cells expressing an endogenous level of DAB2IP 14. The radiation resistance of DAB2IP deficient bladder cancer translates into worse cancer‐specific survival 25. Furthermore, low levels of DAB2IP were detected in a hepatocellular carcinoma subclass from patients with poor survival 26. Taken together, these studies suggest that loss of DAB2IP may portend worse survival in multiple different malignancies.

Enhancer of Zeste homolog 2 (EZH2), a histone lysine methyltransferase, suppresses DAB2IP gene expression by recruiting both polycomb repressor complex and histone deacetylase 1 to the *DAB2IP* promoter region 27. Overexpression of EZH2 has been shown to drive prostate cancer progression, and studies have shown that clinically localized tumors that express high levels of EZH2 have worse outcome 28, 29.

Our pilot study, which analyzed 46 men's diagnostic prostate biopsies for DAB2IP expression and had short follow up, showed that decreased DAB2IP tumor expression correlated with worse clinical outcome in the high‐risk population 13. This same pilot study also analyzed 48 biopsy specimens for EZH2 expression. While increasing expression of EZH2 trended toward worse outcome it was not statistically significant in our high‐risk cohort. This current study attempted to answer our hypothesis that with longer follow up and increased sample size, pretreatment tumor status of DAB2IP would ultimately predict worse prostate cancer‐specific survival (PCSS).

## Material and Methods

### Patient selection

All patients with prostate cancer treated with external beam radiation at the University of Texas Southwestern Medical Center between December 2005 and July 2012 were identified. Of the 658 patients identified, 138 patients met the NCCN guidelines for high‐risk classification (Stage ≥T3a, or Gleason score ≥8, or PSA ≥20) and did not receive any surgical management. Patients with metastases prior to initial radiation therapy (i.e., M1 stage disease) were excluded from the study. All patients were treated definitively with external beam radiotherapy using a variety of techniques, including dynamic arc therapy and intensity modulated radiation therapy (IMRT). All patients were treated with a prostate prescription dose exceeding 72 Gy. Patient data was coded into a secured database according to our institution review board approved study protocol.

Of the patients identified, only those patients who had preserved pretreatment prostate biopsy tissue available and whose biopsies had a sufficient tumor sample available for analysis were included in the study. Each patient in this study underwent transrectal ultrasound‐guided prostate biopsy and the total number of cores obtained ranged from 4 to 33 with 12 being the most common number of cores obtained (*n* = 54). Based on the pathology report, the core with the highest Gleason score was chosen for immunohistochemistry staining. If multiple cores tied for the highest Gleason score, then the core with the highest percentage of neoplastic cells that still had benign normal prostate tissue (i.e., not 100% neoplastic cells) was chosen.

### Specimen characteristics, staining, and quantification

Diagnostic needle biopsies were mounted in paraffin, and 3–4 *μ*m sections were prepared for standard hematoxylin and eosin staining. Standard immunohistochemistry analysis was performed for DAP2IP and EZH2. Immunostaining was performed using the Benchmark XT automated stainer (Ventana, Tuscan, AZ). Briefly, formalin‐fixed, paraffin‐embedded tissue microarray sections were cut at 3–4 *μ*m and air‐dried overnight. The sections were deparaffinized, rehydrated, subjected to heat‐induced epitope retrieval, and then incubated with anti‐EZH2 (clone:SP129, Ventana, prediluted) or anti‐DAB2IP 30 (homegrown, 1:150) primary antibodies. UltraView universal detection system (Ventana) was used for signal detection. The slides were developed using 3‐3′‐diaminobenzidine chromogen and counterstained with hematoxylin. Specific positive and negative (slides incubated without primary antibody) controls were utilized for each run of immunostains and checked for validation of the assay. Controls for anti‐EZH2 antibody included tissue from prostate and breast adenocarcinoma and normal lymph node that are known to have high EZH2 expression. Anti‐EZH2 protocol was standardized according to the directions in the package insert. This antibody is intended for in vitro diagnostic use. Protocol for anti‐DAP2IP was performed as published previously 30.

Tumor and benign prostate tissue stained with each marker were evaluated by an experienced genitourinary pathologist who was blinded to patient clinical information. Each case was evaluated for the extent (percentage of positive cells) and intensity of staining. The average intensity of positive tumor cells was given a grade (G) score: G0, none; G1, weak; G2, intermediate; and G3, strong. DAB2IP positivity was evaluated as cytoplasmic expression. EZH2 was evaluated as nuclear pattern of expression. Compared to DAB2IP expression in the benign ‘control’ prostate tissue surrounding the neoplastic cells, tumor DAB2IP status was categorized as retained (same or stronger expression) or reduced (weaker expression). EZH2 expression was scored as the intensity of staining, G0–G3.

### Study design

The five clinical endpoints in this study were freedom from biochemical failure (FFBF), castration resistance‐free survival (CRFS), distant metastasis‐free survival (DMFS), overall survival (OS), and prostate cancer‐specific survival (PCSS). FFBF was determined using the Phoenix definition, which denotes a PSA increase of ≥2.0 ng/mL above the nadir PSA level 31. CRFS was defined as ≥2 episodes of rising PSA while on standard hormone therapy in the setting of testosterone levels <50 ng/mL, or if patients were started on second‐line therapy as first‐line hormone therapy was deemed to have failed at the clinical oncologist's discretion. DMFS was determined from clinical chart review documenting date of known metastasis by imaging. PCSS was defined as death due to prostate cancer, radiation toxicity, or unknown cause with distant metastasis or castration resistance. Time to each endpoint was calculated from the first day of radiation therapy.

### Statistical analysis

Kaplan–Meier estimates and log‐rank tests were computed to estimate the FFBF, CRFS, DMFS, PCSS, and OS. Univariate Cox regression analyses were conducted to identify significant clinical risk factors that could be significantly associated with each endpoint, including T stage, N stage, Gleason score, pretreatment PSA, total radiation dose, hormone therapy, and duration of hormone therapy. Fisher's exact test was used to evaluate statistical differences among these factors based on the status of the tumor biomarker. Finally, multivariate analysis using a backward model selection was conducted for each factor that met the inclusion criterion *P* < 0.15 by univariate analysis.

## Results

### DAB2IP results

Seventy‐nine patients with high‐risk prostate cancer treated with definitive radiation therapy from 2005 to 2012 met the inclusion criteria and were evaluated. Twenty‐eight percent (22/79) of patients revealed DAB2IP‐reduction while 72% (57/79) retained DAB2IP. The median follow up times were 4.8 years and 5.3 years for patients in the DAB2IP‐reduced group and DAB2IP‐retained group, respectively. Statistical differences in clinicopathological factors include more advanced T stage (*P* = 0.0149), higher pretreatment PSA (*P* = 0.0379), and more aggressive Gleason score (*P* = 0.0048) all within the DAB2IP‐reduced group. There were no significant differences in patient age at diagnosis, N stage, total radiation dose, hormone therapy, or duration of hormone therapy received (see Table 1).

**Table 1:** Comparing clinicopathological factors based on tumor DAB2IP expression

Category	Reduced DAB2IP % (*n*)	Retained DAB2IP % (*n*)	*P*‐value
Sample size, *n*	22	57	N/A
Median follow up in months (range)	63.8 (50.3–85.4)	57.6 (42.1–74.3)	0.3196
Median age in years (range)	66 (63–71)	65 (59–71)	0.3492
T stage			
T1c	4.5 (1)	20.0 (11)	0.0149
T2a‐c	54.5 (12)	67.3 (37)	
T3a‐b	40.9 (9)	12.7 (7)	
N stage			
N0	90.9 (20)	96.4 (54)	0.3150
N1	9.1 (2)	3.6 (2)	
Pretreatment PSA			
<10	9.1 (2)	35.1 (20)	0.0379
10–20	9.1 (2)	12.3 (7)	
>20	81.8 (18)	52.6 (30)	
Gleason score			
6–7	13.6 (3)	29.8 (17)	0.0048
8	13.6 (3)	38.6 (22)	
9–10	72.7 (16)	31.6 (18)	
Total radiation dose			
73.8–79.0 Gy	40.9 (9)	24.6 (14)	0.1745
79.2 Gy	59.1 (13)	75.4 (43)	
Hormone therapy			
Yes	100.0 (22)	89.5 (51)	0.1782
No	0 (0)	10.5 (6)	
Duration of hormone therapy			
4–21 months	30.0 (6)	42.2 (19)	0.4160
24–36 months	70.0 (14)	57.8 (26)	

PSA, prostate‐specific antigen.

**Figure 1:**
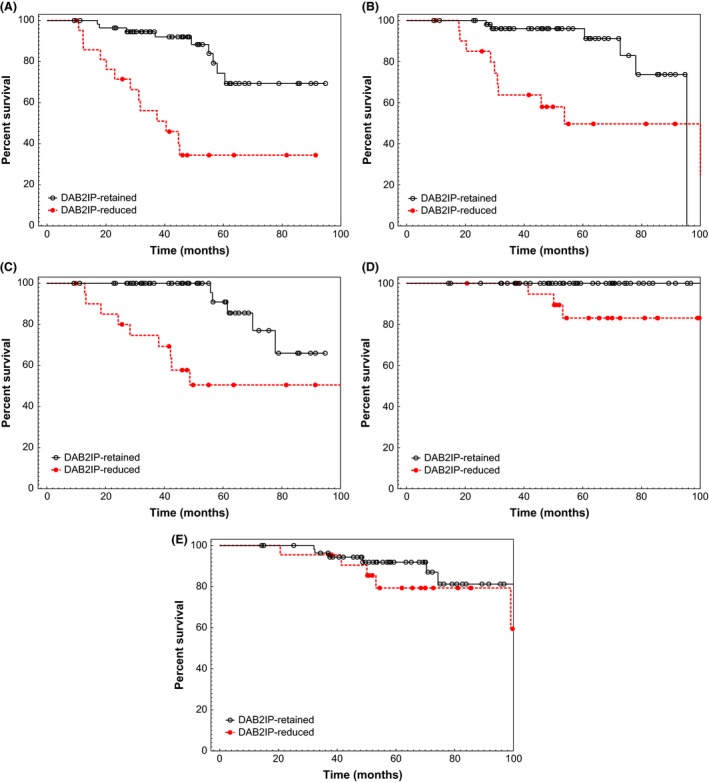
Kaplan–Meier and log‐rank analysis comparing (A) freedom from biochemical failure, (B) castration resistance‐free survival, (C) distant metastasis‐free survival, (D) prostate cancer‐specific survival, and (E) overall survival based on pretreatment tumor DAB2IP status.

Pretreatment tumor reduction in DAB2IP as evidenced by prostate biopsy samples correlated strongly with worse outcome in every endpoint measured except death from any cause (see Table 2). Reduced DAB2IP portended a significantly worse FFBF (*P* < 0.0001), CRFS (*P* = 0.0039), DMFS (*P* = 0.0006), and PCSS (*P* = 0.0102). Kaplan–Meier estimates of these four significant endpoints can be seen in Figure 1. By 4‐years postradiotherapy, the biochemical failure rate was a staggering 66% in the DAB2IP‐reduced group compared to only 8% in the DAB2IP‐retained group at that point. By 5‐years postradiotherapy, the prostate cancer‐specific mortality rate was 17% in the DAB2IP‐reduced group whereas no one in the DAB2IP‐retained group had died due to their malignancy.

**Table 2:** Pretreatment tumor reduction in DAB2IP portends significantly worse outcome as calculated by log‐rank

Biomarker status	FFBF		CRFS		DMFS		PCSS		OS	
	4 years	*P*‐value	4 years	*P*‐value	4 years	*P*‐value	5 years	*P*‐value	5 years	*P*‐value
DAB2IP‐retained	92%	<0.0001	96%	0.0039	100%	0.0006	100%	0.0102	92%	0.3327
DAB2IP‐reduced	34%		58%		58%		83%		79%	

CRFS, castration resistance‐free survival; DMFS, distant metastasis‐free survival; FFBF, freedom from biochemical failure; PCSS, prostate cancer‐specific survival.

Sixteen of the 79 high‐risk patients in the DAB2IP cohort had met the criteria for castration resistance. Thirteen of these patients had testosterone data available to confirm that the PSA continued to rise despite a testosterone level <50 ng/mL. The median testosterone level for these 13 patients was 3 ng/mL and the levels ranged from undetectable to 32 ng/mL. The remaining three patients were determined to have clinically become castration resistant because of a rising PSA despite adding a second agent to their androgen deprivation therapy.

### EZH2 results

Ninety‐seven patients with high‐risk prostate cancer treated with definitive radiation therapy from 2005 to 2012 met the inclusion criteria and were evaluated. Ninety‐eight percent (95/97) of patients expressed EZH2 as follows: 2% (2/97) were G0, 10% (10/95) were G1, 68% (66/97) were G2, and 20% (19/97) were G3. The median time to follow up was 4.6 years in the EZH2 cohort. Stratified EZH2 expression was not statistically significant for any outcome (see Table 3).

**Table 3:** Stratified EZH2 expression was not statistically significant for any outcome as calculated by log‐rank

Biomarker status	FFBF		CRFS		DMFS		PCSS		OS	
	4 years	*P*‐value	4 years	*P*‐value	4 years	*P*‐value	5 years	*P*‐value	5 years	*P*‐value
EZH2 grade 0	100%	0.6324	100%	0.1125	100%	0.2536	100%	0.1715	100%	0.2432
EZH2 grade 1	90%		89%		90%		100%		80%	
EZH2 grade 2	75%		87%		85%		95%		89%	
EZH2 grade 3	69%		69%		75%		81.8%		77%	

CRFS, castration resistance‐free survival; DMFS, distant metastasis‐free survival; FFBF, freedom from biochemical failure; PCSS, prostate cancer‐specific survival.

Nine patients in the EZH2 study elected to not receive hormone therapy. Of these, one patient did not express EZH2, one patient had G1 expression, and the remaining seven patients all had G2 expression. Importantly, none of these nine patients died from any cause let alone from their malignancy. Furthermore, even though every patient with the highest expression of EZH2 (G3) received androgen deprivation, these patients demonstrated the worst outcomes at 4 years for FFBF, CRFS, and DMFS.

### Univariate and multivariate analysis

Univariate analysis using Cox regression of typical prognostic variables revealed that only T stage, N stage, and Gleason score correlated with outcome. Higher T stages were significantly associated with worse FFBF (*P* = 0.0477), CRFS (*P* = 0.0379), DMFS (*P* = 0.0082), and PCSS (*P* = 0.0105). N1 stage was significantly associated with worse FFBF (*P* = 0.0003), CRFS (*P* = 0.0132), and DMFS (*P* < 0.0001) but not PCSS (*P* = 0.2782). Higher Gleason scores were significantly associated with worse FFBF (*P* = 0.0007), CRFS (*P* = 0.0043), DMFS (*P* = 0.0083), and PCSS (*P* = 0.0031). None of the prognostic variables were significantly associated with OS.

Table 4 shows all variables that were significant for each outcome after multivariable analysis. Pretreatment tumor DAB2IP status remained significant in the multivariable analysis for FFBF (*P* = 0.0026), CRFS (*P* = 0.0043), and DMFS (*P* = 0.0009). The two other significant variables on multivariable analysis include Gleason score (FFBF *P* = 0.0428; CRFS *P* = 0.0409; DMFS *P* = 0.0174) and T stage (DMFS *P* = 0.0252). Multivariate analysis could not be conducted for PCSS because zero patients had died due to prostate cancer in the subgroups of retained‐DAB2IP, T1c‐T2b stages, N0 stage, or Gleason scores 6–8. Interestingly, not only was DAB2IP significant for each measureable outcome after multivariable analysis, but it was always the most highly significant variable.

**Table 4:** Statistically significant variables on multivariate analysis by endpoint as calculated by Cox regression

Freedom from biochemical failure		
DAB2IP	*P* = 0.0026	HR = 3.994
Gleason score	*P* = 0.0428	HR = 2.674
Castration resistance‐free survival		
DAB2IP	*P* = 0.0043	HR = 6.614
EZH2	*P* = 0.0566	HR = 9.615
Gleason score	*P* = 0.0409	HR = 4.274
Distant metastasis‐free survival		
DAB2IP	*P* = 0.0009	HR = 12.076
EZH2	*P* = 0.0160	HR = 25.641
Gleason score	*P* = 0.0174	HR = 6.049
T‐stage	*P* = 0.0252	HR = 4.717

## Discussion

DAB2IP is a putative tumor suppressor and member of the RAS‐GAP family. Decreased expression of the protein DAB2IP is associated with aggressive disease 12, 13 and radiation resistance 9, 14, 32, 33. Downregulation of DAB2IP has mostly been attributed to epigenetic regulation involving methylation of the promoter region mediated by EZH2 17, 18, though Skp2‐mediated proteasome degradation 23 and genetic variants (i.e., mutations) 12, 34, 35 may also play a role. Decreased DAB2IP has been identified in several cancers other than prostate, including breast 19, 36, hepatocellular 20, 26, lung 21, gastrointestinal 22, and bladder 14, 24.

Based on strong preclinical data indicating the role of DAB2IP in radiation response in prostate cancer cells, we performed a pilot study to determine its clinical importance 13. Our prior pilot study analyzed 46 men's diagnostic prostate biopsies with high‐risk disease for DAB2IP expression and showed a strong correlation between pretreatment tumor DAB2IP reduction and worse clinical outcome with only 2.7 years of median follow up. With longer follow up and increased sample size, we hypothesized that pretreatment tumor status of DAB2IP would also predict worse malignancy‐specific survival (PCSS). In addition to studying DAB2IP, we also sought to determine the potential prognostic role of its upstream regulator, EZH2, after increasing the sample size and follow up time.

In this study we show that pretreatment tumor DAB2IP status and not EZH2 correlates with worse rates of biochemical failure, castration resistance, and distant metastasis after the standard of care treatment for these high‐risk prostate cancer patients including definitive radiation therapy and androgen deprivation. Additionally, our results confirmed our hypothesis that DAB2IP reduction ultimately leads to significantly increased mortality due to prostate cancer. Five of the six patients (83%) in this study that died due to their malignancy had an initial Eastern Cooperative Oncology Group (ECOG) score of 0 and therefore performance status did not confound our results. Based on the numerical data included in the opening introductory paragraph of this manuscript and our observation that about one‐fourth of high‐risk prostate cancer patients have decreased expression of DAB2IP, we estimate that between 12,000 and 18,000 men treated annually in the United States have suboptimal response to the current standard of care treatment.

Another important finding from this study is that the tumors with reduced DAB2IP expression were statistically more likely to have a more advanced T stage, higher pretreatment PSA, and more aggressive Gleason score. Whether down‐regulation of DAB2IP is the molecular cause of these more aggressive clinicopathologic findings or whether the decreased DAB2IP expression selectively occurs in aggressive tumors remains unclear.

Nearly all high‐risk prostate cancer samples in this study expressed EZH2. Rather than predict cancers with poor therapeutic response, EZH2 levels may help screen for higher risk cancers. Nevertheless, it's interesting to note that tumors with the highest expression of EZH2 (G3) demonstrated the worst outcomes at 4 years for FFBF, CRFS, and DMFS, which is congruent with the idea that increased EZH2 leads to methylation of the *DAB2IP* promotor and reduction in DAB2IP expression. Since regulation of DAB2IP expression is not the sole function of EZH2, it makes sense that it would not be as potent of a prognostic biomarker compared to DAB2IP which modulates different signal cascades associated with cell proliferation, survival, apoptosis, and metastasis.

Based on the results presented herein, DAB2IP may be able to differentiate the worst cases amongst the already high‐risk group of prostate cancer patients that are at greatest risk for treatment failure prior to initiation of standard therapy. Potential clinical application is that in the future we may be able to effectively use tumor DAB2IP expression from a patient's biopsy specimen as a means of determining curability of their cancer with standard radiation‐based therapeutic regimens.

Future research may be directed toward creating molecularly based therapeutic strategies to upregulate DAB2IP and restore the tumor suppressor's presence within the neoplastic cells or by targeting downstream effectors. DNA methyltransferase inhibitors as well as histone deacetylase inhibitors can both induce expression of the *DAB2IP* gene 17, 18. Cytolethal distending toxin (CTD) from Campylobacter jejuni significantly elicited cell death in DAB2IP‐deficient prostate cancer cells when combined with radiotherapy 37. Restoring DAB2IP may also improve castration resistance in addition to radiation resistance as DAB2IP expression inversely correlates with androgen receptor activation status particularly in recurrent or metastatic prostate cancer patients 16.

The DAB2IP pathway is an important potential target for improving the treatment of multiple malignancies (not just prostate cancer) and enhancing multiple modalities (not just radiation therapy). For example, KU55933 which suppresses ATM phosphorylation upon irradiation could be applied in the radiotherapy of bladder cancer patients with a DAB2IP gene defect 14. Furthermore, other than improving radiation responsiveness, targeting the DAB2IP pathway may also improve response to chemotherapy 33. Inhibiting EZH2 through siRNA has been shown to increase DAB2IP expression 27 and reverse chemoresistance 38. Improving chemotherapy response could improve survival in this population as patients with reduced DAB2IP in bladder cancer treated with surgery and adjuvant chemotherapy had worse cancer‐specific survival 24.

One limitation of this study includes the way in which tumor DAB2IP status is characterized as reduced or retained. We were not able to identify a specific intensity level threshold below which DAB2IP should be considered reduced, especially since some samples stained better than others with the antibody. Instead, we compared the tumor staining with the surrounding normal prostate tissue as the control to know whether the tumor retained normal DAB2IP expression or reduced the protein's expression. The limitation of this definition comes when the entire stained sample is tumor and there are no benign glands with which to compare, or when there are very few glands with which to compare and they are all atrophic with high DAB2IP expression. Perhaps this problem could be avoided via a prospective study using fresh tissue and not relying on a retrospective study with tissues at varying ages.

Another limitation of this study is not having a separate cohort of patients that received a nonradiation intervention such as prostatectomy with which to compare. At this point, we do not definitively know whether DAB2IP reduction is a poor prognostic indicator regardless of the intervention, or if it really is a marker of radiation resistance. Additionally, since the tissue specimens were obtained from biopsy, there is a clear limitation of potential heterogeneity of pathological findings and the immunostaining not always reflecting the characteristics of all cancer foci in each patient. Also, even though we were able to increase the sample size by over 70% from our prior study and obtain over two more years of additional follow up data, the sample size and follow up time remain limitations. DAB2IP requires further studies to validate its role in this cohort of patients and to further determine whether it may also be a predictive marker of response to radiation therapy.

In conclusion, based on our results about one‐fourth of men with high‐risk prostate cancer and well over 10,000 men in the United States annually may have reduced tumor expression of DAB2IP which makes their tumors more radioresistant and aggressive. This subpopulation with reduced DAB2IP has a suboptimal response and significantly higher prostate cancer‐specific mortality despite standard of care treatment with radiation therapy and androgen deprivation. Targeting the DAB2IP pathway may be a promising area for future drug development to improve the effectiveness of radiation and chemotherapy in multiple malignancies. Further study into improving the radiation response and survival in this subpopulation is warranted.

## Conflict of Interest

There are no conflicts of interest.
